# Prognostic Analysis of the IDH1 G105G (rs11554137) SNP in IDH-Wildtype Glioblastoma

**DOI:** 10.3390/genes13081439

**Published:** 2022-08-12

**Authors:** Ayoub Saaid, Matteo Monticelli, Alessia Andrea Ricci, Giulia Orlando, Cristina Botta, Pietro Zeppa, Andrea Bianconi, Simona Osella-Abate, Francesco Bruno, Alessia Pellerino, Roberta Rudà, Paola Cassoni, Diego Garbossa, Fabio Cofano, Luca Bertero

**Affiliations:** 1 Neurosurgery Unit, Department of Neuroscience “Rita Levi Montalcini”, University of Turin, 10126 Turin, Italy; 2 Neurosurgery Unit, Department of Neuroscience and Rehabilitation, University of Ferrara, 44100 Ferrara, Italy; 3 Pathology Unit, Department of Medical Sciences, University of Turin, 10126 Turin, Italy; 4 Pathology Unit, Department of Oncology, University of Turin, 10126 Turin, Italy; 5 Neuro-Oncology Unit, Department of Neuroscience “Rita Levi Montalcini”, University of Turin, 10126 Turin, Italy; 6 Department of Neurology, Castelfranco and Treviso Hospitals, 31100 Treviso, Italy

**Keywords:** IDH1, glioblastoma, rs11554137, SNP, prognosis

## Abstract

The G105G SNP (rs11554137) in the IDH1 gene is observed in about 10–15% of patients with a diffuse glioma. Data regarding its impact on gliomas are poor and partially conflicting, possibly due to the evolving classification of CNS tumors. The aim of this study was to investigate the G105G SNP prognostic significance in a homogenous cohort of IDH-wildtype glioblastomas, in agreement with the 2021 WHO classification. The study analyzed 211 patients by collecting several clinico-pathological and molecular characteristics, including the age, lesion localization, number of involved lobes, type of surgical treatment, disease outcome and MGMT promoter methylation status. PFS and DSS curves were plotted according to the Kaplan–Meier method and statistical analyses were performed using parametric and non-parametric tests. A total of 32 patients out of 211 (15.2%) were found to be G105G SNP carriers. No significant impact of the IDH1 G105G SNP on patients’ outcomes was observed in terms of PFS and DSS, while MGMT promoter methylation and gross total resection resulted as key prognostic factors in our cohort as expected. No prognostic impact of the IDH1 G105G SNP was detected in this strict cohort of IDH-wildtype glioblastomas. Analysis of larger cohorts is warranted to address the sample size limitations.

## 1. Introduction

Isocitrate dehydrogenase (IDH) mutational status is a strong prognostic criterion affecting the natural history of central nervous system (CNS) diffuse gliomas; this was clearly underlined in the 2016 World Health Organization (WHO) classification of CNS tumors and further stressed in the 2021 edition [1,2]. IDH-mutant diffuse gliomas represent biologically distinct neoplasms compared to the IDH-wildtype glioblastoma and, for this reason, the WHO 2021 classification restricted the use of the glioblastoma term to this entity, replacing the previous IDH-mutant glioblastoma with the novel astrocytoma, IDH-mutant, grade 4. IDH1/IDH2 mutations are also present in other neoplasms, such as acute myeloid leukemia (AML), chondrosarcoma and cholangiocarcinoma [3]. IDH1/IDH2 mutations are detected in about 10% of AML cases but, differently from diffuse gliomas, the prognostic role of this molecular hallmark is controversial in this different neoplasm, although it represents an effective therapeutic target [4].

Concerning the IDH1 gene, a single nucleotide polymorphism, rs11554137: C > T of exon 4, codon 105 (G105G SNP), is observed in about 10% of cases. The G105G single-nucleotide polymorphism is a synonymous polymorphism (Glycine > Glycine) located in exon 4 of the IDH1 gene. Interestingly, this genetic site is the region where the most frequent somatic IDH1 mutation occurs: the R132H mutation. Acquaviva G et al. investigated the prevalence of the G105G SNP and found this polymorphism to be three-fold more frequent in patients diagnosed with infiltrating gliomas compared to the general population [5].

The prognostic significance of the G105G SNP was initially assessed in AML, demonstrating its independent association with worse prognosis in normal karyotype myeloid leukemias (AML-NK) [6,7,8]. Few studies have explored the prognostic role of the G105G SNP in diffuse gliomas [9,10,11], with conflicting data. Wang X et al. initially reported an unfavorable prognostic significance of the G105G SNP in malignant gliomas, while following studies did not observe this association. Conversely, Mistry AM et al. found no survival difference among patients with glioblastoma, according to the G105G SNP presence. These differences could be, at least, partially due to the evolving classification of CNS tumors during the last few years and the mixing of IDH-mutant and IDH-wildtype cases.

No conclusive evidence of the G105G SNP’s potential biological role has been acquired to date. In gliomas, its occurrence seems to be independent of IDH1/IDH2 somatic mutations, while an inverse correlation with EGFR amplification has been suggested, but not confirmed, in an independent validation series [5,9]. In acute myeloid leukemia, the G105G SNP has shown a certain degree of mRNA stability interference [6]. Based on sequence prediction analysis, it has been hypothesized that codon 105 of the IDH1 gene might be part of an exonic splicing silencing (ESS) site motif [12]. Furthermore, studies regarding synonymous polymorphisms’ role in disease physiopathology suggested that they could alter protein folding, interfere with mRNA stability and hamper constitutive or alternative splicing [13].

The aim of the present study was to investigate whether the G105G SNP has a prognostic role, focusing on glioblastoma considering IDH-wildtype samples only, as per the recent 2021 WHO classification of CNS tumors.

## 2. Materials and Methods

This retrospective single-center study included patients with a diagnosis of IDH-wildtype glioblastoma, surgically treated at the Neurosurgery Unit, Dept. of Neuroscience ‘‘Rita Levi Montalcini”, diagnosed at the Pathology Unit, Dept. of Medical Sciences, and managed for the adjuvant treatments and follow-up at the Neuro-Oncology Unit of the “Città della Salute e della Scienza” University Hospital of Turin, between 2016 and 2018.

Inclusion criteria were as follows: (1) histopathological diagnosis of glioblastoma (GBM), IDH-wildtype, according to the WHO 2021 classification of CNS tumors. Concerning this criterion, according to the 2021 WHO classification, grade 2 and grade 3 IDH-wildtype astrocytomas must be considered de facto glioblastomas when EGFR gene amplification and/or TERT promoter mutation and/or both whole chromosome 7 gain and chromosome 10 loss are present. However, although the poor outcome of these so-called molecular glioblastomas has been well acknowledged, data suggest a slightly more favorable outcome for these patients compared to IDH-wildtype glioblastomas harboring canonical morphological features (microvascular proliferation and/or necrosis) [14]. For this reason, and in consideration of their rarity, we excluded these cases; (2) molecularly proven (by Sanger sequencing or next-generation sequencing) IDH1/IDH2 wildtype status; (3) age >18 years; (4) informed consent.

Exclusion criteria were: (1) insufficient material for molecular analysis; (2) presence of H3 K28 (K27) mutation in midline cases; (3) spinal tumor location.

The study was conducted in accordance with the Code of Ethics of the World Medical Association (Declaration of Helsinki and following amendments) for experiments involving humans and within the guidelines and regulations defined by the University of Turin.

Methyl-guanosine methyl transferase (MGMT) promoter methylation status has been analyzed by pyrosequencing, using a ≥9% average methylation level of CpG islands to define the methylated cases, according to Dunn J et al. [15]. Then, we stratified the methylated samples into two groups (9–29% and ≥30%).

The CNS neoplasm’s location was divided into: hemispheric, for cortical/subcortical located gliomas distinguishing the number of involved lobes, and midline, for brainstem-, cerebellar-, thalamus- and hypothalamus-located neoplasms. Multifocality, defined as at least two radiologically separate contrast-enhanced nodes without FLAIR alteration between them, was also considered. Concerning surgery, we identified three groups: those who had (i) biopsy surgery, whether it was stereotactic or open; (ii) partial/subtotal surgery; or (iii) gross total resection (GTR) surgery in patients with no residual tumor, neither intra-operatively nor at the postoperative (<48-h) magnetic resonance imaging (MRI).

The disease progression (PD) was clinically and radiologically evaluated according to the RANO criteria, based on MRI reports [16]; we did not distinguish early PD (pre-adjuvant treatments) from late PD (post-adjuvant treatments), excluding the possibility of pseudoprogression. The progression pattern was classified as local and distant, and also by assessing the presence of leptomeningeal dissemination.

Progression-free survival (PFS) was calculated from the diagnosis date to PD or until the last follow-up. Disease-specific survival (DSS) was defined as the interval from the diagnosis date to death, considering death by any cause. Data were collected from patients’ clinical files.

The differences in the variables’ distribution were analyzed using parametric and non-parametric tests (the Student’s *t*-test, Pearson’s chi-squared test, and Wilcoxon rank test). To identify the clinical and/or molecular factors related to PFS and DSS, survival curves were plotted according to the Kaplan–Meier method, and differences between the curves were assessed using the Mantel log-rank test. The assumptions of probability, according to the Cox model, were subsequently analyzed with the Schoenfeld residual proportional hazards test.

## 3. Results

### 3.1. IDH1 G105G SNP and Clinical/Pathological Characteristics

According to the inclusion/exclusion criteria, we collected 211 patients. Of those, 32 patients (15.2%) were found to be IDH1 G105G SNP carriers. In this study, we considered several clinical and pathological characteristics, such as age, lesion localization, number of involved lobes, type of surgical treatment (biopsy, partial and gross total resection) and disease outcome. The group of the G105G SNP carriers and the group of non-carriers have been compared. There was no statistical difference between the two groups for any of the above-mentioned features, as shown in Table 1 and Table 2. The median age of both groups was in the seventh decade of life. There was no significant difference in prevalence of the G105G SNP between the male and female patients. Concerning the molecular characteristics, the prevalence of MGMT methylation was not statistically different between the two groups (*p* = 0.594).

#### 3.1.1. Progression-Free Survival (PFS) Analysis

**Figure 1:**
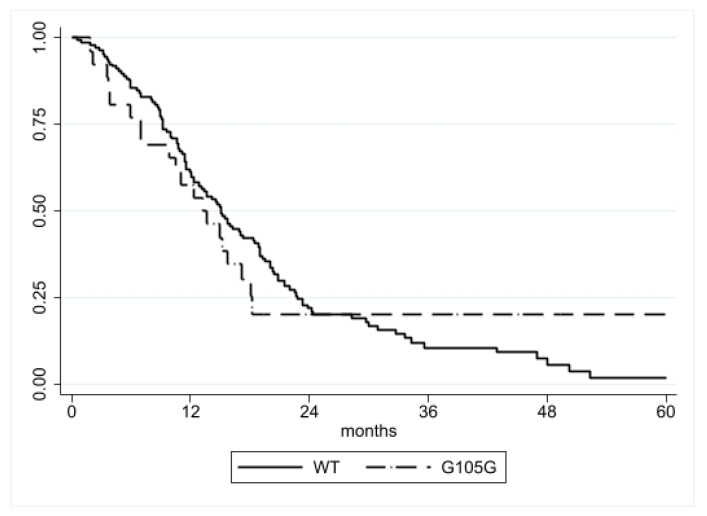
PFS according to IDH1 G105G status. No significant difference was detected (*p* = 0.9770).

Data about disease progression were available in 172/211 (81.5%) patients. The median PFS was similar in patients with the IDH1 G105G SNP (13.3 months; 25th–75th: 7.0–18.2) compared to patients without it (15.0 months; 25th–75th: 9.2–22.8), and no significant difference was found by log-rank analysis (*p* = 0.9770) (Figure 1). Similarly, Cox regression analysis did not show any association with PFS (HR = 1.01, CI = 0.62–1.62, *p* = 0.977) (Table 3).

Several other clinical and molecular features correlated with time to disease progression, including higher MGMT promoter methylation, which was significantly associated with a more favorable PFS (9–29%: HR = 0.44, CI = 0.26–0.76, *p* = 0.003; ≥30%: HR = 0.54, CI = 0.37–0.79, *p* = 0.001). The gross total surgery group also showed a trend towards longer PFS, but it did not reach statistical significance (HR = 0.61, CI = 0.37–1.01, *p* = 0.058) (Table 3).

Conversely, cerebellar localization (HR = 41.5, CI = 4.64–371, *p* = 0.001), the involvement of 3 lobes (HR = 2.37, CI = 1.31–4.28, *p* = 0.004) and a multifocal growth pattern (HR = 2.37, CI = 1.43–3.90, *p* = 0.001) showed an adverse effect on PFS, but the sample size of cerebellar neoplasms was remarkably limited and, thus, this finding should be cautiously interpreted.

#### 3.1.2. Disease Specific Survival (DSS) Analysis

**Figure 2:**
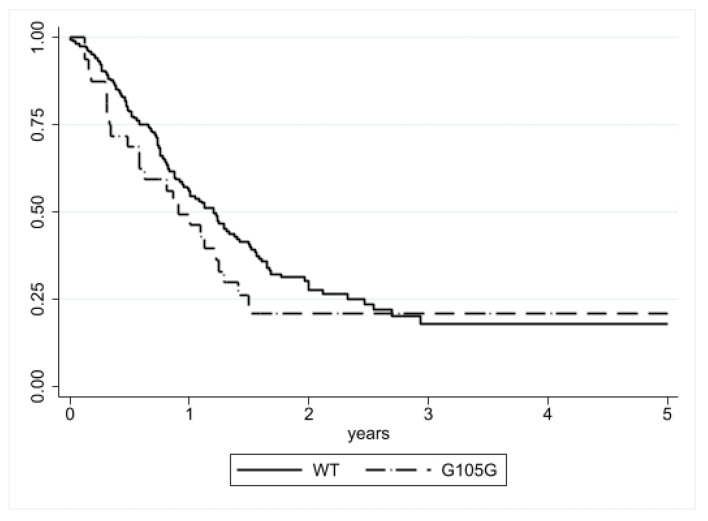
DSS according to IDH1 G105G status. No significant difference was detected (*p* = 0.1833).

The median DSS was similar in patients with (0.90 years; 25th–75th: 0.33–1.49) and without (1.20 years; 25th–75th: 0.65–2.46) the IDH1 G105G SNP, and no statistical difference was observed with the log-rank test (*p* = 0.1833) (Figure 2). Cox regression analysis also did not observe any difference (HR = 1.34, CI = 0.87–2.08, *p* = 0.185) (Table 4).

Concerning the other variables, gross total surgery proved to be a favorable factor for DSS (HR = 0.53, CI = 0.33–0.8, *p* = 0.010) (Table 4), as well as MGMT promoter methylation status (9%–29%: HR = 0.53, CI = 0.32–0.89, *p* = 0.016; ≥30%: HR = 0.43, CI = 0.28–0.64, *p* = <0.001). Conversely, cerebellar localization (HR = 5.55, CI = 1.35–22.9, *p* = 0.018), involvement of 3 lobes (HR = 2.28, CI = 1.31–4.0, *p* = 0.004) and multifocality (HR = 2.25, CI = 1.38–3.66, *p* = 0.001) showed an unfavorable prognostic significance as observed for PFS.

## 4. Discussion

In the present series, 32 patients out of 211 (15.2%) were found to be G105G SNP carriers. This result is in line with previous studies regarding this polymorphism prevalence. In the series analyzed by Wang X et al. [9], the rates of the G105G SNP carriers varied between 8% and 11.4%, with no association between the tumor grade and SNP prevalence. Acquaviva G et al. [5] observed an overall SNP prevalence of 15% among patients with brain tumors and, differently from the previous study, the carrier rate varied according to the tumor grade, with the highest prevalence found among grade 3 tumors (26.1%) and lower rates in grade 4 (13.7%) and 2 (10.9%) neoplasms. However, this study was not limited to diffuse gliomas, but included also other types of brain tumors, such as ependymomas. Mistry AM et al. [10] evaluated a series of 171 glioblastomas (both IDH-mutant and IDH-wildtype), observing 16 (9.4%) G105G SNP carriers. More recently, Franceschi E et al. [11] analyzed a series of IDH-mutant grade 2 and 3 diffuse gliomas, detecting a 12.7% prevalence.

The second issue to be considered is the association between SNP presence and other clinical, pathological or molecular characteristics. The more comprehensive analysis was performed by Wang X et al., who observed no significant association with IDH1/IDH2 mutations and MGMT promoter methylation status; an inverse correlation between IDH1 G105G SNP presence and EGFR amplification was found in the first analyzed series, but this finding was not confirmed in a second independent cohort [9]. Acquaviva G et al. found a higher prevalence of the IDH1 G105G SNP in patients with grade 2 and 3 IDH-wildtype tumors (43.8%) compared to IDH-mutant tumors (11.5%) (*p* = 0.005) [5]. Finally, Mistry AM et al. found no associations between the presence of the IDH1 G105 SNP and other variables [10]. Our study was focused on IDH-wildtype glioblastoma, so it was not possible to analyze the association with IDH mutational status, but we explored the relationship with clinical features as well as MGMT promoter methylation status, observing no significant associations.

A further and arguably more important question is whether the IDH1 G105G harbors a prognostic significance in patients with diffuse gliomas. In the first series analyzed by Wang X et al. [9], reduced PFS and OS were reported in GBM (PFS: 6.4 months vs. 8.5 months, *p* = 0.003; OS: 10.7 months vs. 15.5 months, *p* = 0.001). This prognostic relevance was confirmed in an independent series of 306 GBM, but it did not reach significance in a further series of 337 GBM. Finally, it should be noted that these GBM series included both IDH-mutant and IDH-wildtype tumors.

Mistry AM et al. [10] analyzed a series of 171 GBM, which also included 7 IDH-mutant GBM. G105G SNP carriers showed a similar outcome compared to other IDH-wildtype glioblastoma patients (OS: HR = 0.82, CI = 0.45–1.49, *p* = 0.55; PFS: HR = 0.69, CI = 0.40–1.21, *p* = 0.360).

More recently, Franceschi E et al. [11] observed a favorable prognostic impact of IDH1 G105G SNP in a series of IDH-mutant grade 2/3 gliomas (PFS: not reached vs. 47.3 months, *p* = 0.015); multivariate analysis confirmed this finding (HR 0.240; CI = 0.074–0.784, *p* = 0.018), but the main limitation of this study is the limited number of patients (71 patients, 9 SNP carriers), which precludes any firm conclusion.

To address these inconsistencies and to comply with the WHO 2021 diagnostic criteria, we focused our analysis on a homogenous cohort of IDH-wildtype glioblastomas. Our results show no significant impact of IDH1 G105G SNP on patients’ outcome in terms of PFS and DSS, supporting the findings by Mistry AM et al. [10].

The main limitations of the present study are the retrospective collection of data, the lack of a more comprehensive molecular profiling and of a more granular stratification of adjuvant treatments, which could have allowed us to verify the potential association of the IDH1 G105G SNP with other molecular/clinical traits.

Finally, MGMT promoter methylation and gross total resection (GTR) resulted as key prognostic factors in our cohort, in line with the consolidated literature data [17,18].

## 5. Conclusions

In the present study, no association between the IDH1 G105G SNP and the prognosis was detected in a strict cohort of IDH-wildtype glioblastoma. Although it is possible that sample size limitations could have hampered the capability to detect a small effect on the prognosis, the clinical relevance of such a potential association is expected to be limited. Future meta-analyses or multicentric studies are warranted to investigate this possibility.

## Figures and Tables

**Table 1:** Clinical characteristics according to IDH1 G105G SNP status.

		Total (*n* = 211)	G105G IDH		
			NO	YES	*p*
**Age**	Median	66	66	64	0.868
	Interval	23–84	23–84	32–80	
	<55	47 (22.3%)	40 (85.1%)	7 (14.9%)	0.953
	>55	164 (77.7%)	139 (84.7%)	25 (15.3%)	
**Sex**	F	80 (37.9%)	65 (81.2%)	15 (18.8%)	0.257
	M	131 (62.1%)	114 (87.0%)	17 (13.0%)	
**Tumor site (missing: 2)**	Hemispheric	200 (95.7%)	170 (85.0%)	30 (15.0%)	0.679
	Midline	6 (2.9%)	5 (83.3%)	1 (16.7%)	
	Cerebellar	3 (1.4%)	2 (66.7%)	1 (33.3%)	
**Main involved lobe** **(missing: 11)**	Frontal	48 (24.0%)	42 (87.5%)	6 (12.5%)	0.792
	Temporal	49 (24.5%)	42 (85.7%)	7 (14.3%)	
	Parietal	13 (6.5%)	12 (92.3%)	1 (7.7%)	
	Occipital	81 (40.5%)	66 (81.5%)	15 (18.5%)	
	No predominant lobe	9 (4.5%)	8 (88.9%)	1 (11.1%)	
**Number of** **involved lobes (missing: 12)**	1	119 (59.8%)	104 (87.4%)	15 (12.6%)	0.486
	2	60 (30.2%)	49 (81.7%)	11 (18.3%)	
	3	20 (10.1%)	16 (80.0%)	4 (20.0%)	
**Multifocal** **tumor**	No	190 (90.1%)	163 (85.8%)	27 (14.2%)	0.245
	Yes	21 (10.0%)	16 (76.2%)	5 (23.8%)	
**Type of surgery**	Biopsy	24 (11.4%)	19 (79.2%)	5 (20.8%)	0.448
	Partial	74 (35.0%)	61 (82.4%)	13 (17.6%)	
	Gross	113 (53.6%)	99 (87.6%)	14 (12.4%)	
**Progression (missing: 39)**	No	33 (19.2%)	28 (84.8%)	5 (15.2%)	0.995
	Yes	139 (80.8%)	118 (84.9%)	21 (15.1%)	
**Type of** **progression (missing: 73)**	Local	116 (84.0%)	98 (84.5%)	18 (15.5%)	0.284
	Distant	2 (1.4%)	1 (0.5%)	1 (0.5%)	
	Local + Distant	17 (12.3%)	16 (94.1%)	1 (5.9%)	
	Leptomeningeal dissemination	3 (2.2%)	2 (66.7%)	1 (33.3%)	
**Outcome at last follow-up**	Alive	71 (33.6%)	64 (90.1%)	7 (9.9%)	0.126
	Deceased	140 (66.4%)	115 (82.1%)	25 (17.9%)	

Significance threshold: *p* < 0.05.

**Table 2:** Pathological and molecular characteristics according to IDH1 G105G SNP status.

		Total (*n* = 211)	G105G IDH		
			NO	YES	*p*
**MGMT** **(missing: 3)**	Median	8	8	6	0.594
	Interval	1–81	1–81	1–61	
**MGMT** **promoter methylation status** **(missing: 3)**	<9%	108 (51.9%)	90 (83.3%)	18 (16.7%)	0.324
	9–29%	33 (15.9%)	26 (78.8%)	7 (21.2%)	
	≥30%	67 (32.2%)	60 (89.6%)	7 (10.4%)	
**Mitotic count**	Median	11	11	14	0.672
	Interval	2–72	2–72	4–51	
**Ki-67**	Median	30	30	27	0.120
	Interval	5–90	5–90	15–75	

Significance threshold: *p* < 0.05.

**Table 3:** PFS analysis of clinical/pathological/molecular features by Cox regression.

		HR ^1^	CI	*p*
**IDH1 G105G SNP**	Present vs. Absent	1.01	0.62–1.62	0.977
**Sex**	M vs. F	1.13	0.81–1.61	0.460
**Age**	Linear	1.01	1.00–1.03	**0.008**
	>55 vs. <55	1.41	0.92–2.12	0.106
**MGMT promoter** **methylation status**	<9%	1		
	9–29%	0.44	0.26–0.76	**0.003**
	≥30%	0.54	0.37–0.79	**0.001**
**Mitotic count**	Linear	1.01	0.46–1.85	0.813
**Tumor site**	Hemispheric	1		
	Midline	0.60	0.22–1.64	0.324
	Cerebellar	41.5	4.64–371	**0.001**
**Main involved lobe**	Frontal	1		
	Temporal	0.58	0.36–0.93	**0.025**
	Parietal	0.51	0.23–1.16	0.111
	Occipital	0.84	0.54–1.30	0.431
	No predominant lobe	1.60	0.70–3.62	0.261
**Number of** **involved lobes**	1	1		
	2	0.95	0.64–1.41	0.789
	3	2.37	1.31–4.28	**0.004**
**Multifocal tumor**	Yes vs. No	2.37	1.43–3.90	**0.001**
**Surgery type**	Biopsy	1		
	Partial	1.10	0.64–1.87	0.729
	Gross	0.61	0.37–1.01	0.058

Significance threshold: *p* < 0.05. ^1^ HR = Hazard Ratio, CI = Confidence Interval.

**Table 4:** DSS analysis of clinical/pathological/molecular features by Cox regression.

		HR ^1^	CI	*p*
**IDH1 G105G SNP**	Present vs. Absent	1.34	0.87–2.08	0.185
**Sex**	M vs. F	1.07	0.76–1.51	0.704
**Age**	Linear	1.01	0.99–1.03	0.056
	>55 vs. <55	1.31	0.87–1.97	0.189
**MGMT promoter** **methylation status**	<9%	1		
	9–29%	0.53	0.32–0.89	**0.016**
	≥30%	0.43	0.28–0.64	**<0.001**
**Mitotic count**	Linear	1.00	0.98–1.02	0.858
**Tumor site**	Hemispheric	1		
	Median line	0.76	0.28–2.08	0.604
	Cerebellar	5.55	1.35–22.9	**0.018**
**Main involved lobe**	Frontal	1		
	Temporal	0.57	0.34–0.95	**0.030**
	Parietal	0.74	0.36–1.51	0.411
	Occipital	0.97	0.63–1.49	0.876
	No predominant lobe	1.03	0.40–2.64	0.953
**Number of** **involved lobes**	1	1		
	2	1.09	0.74–1.61	0.645
	3	2.28	1.31–4.00	**0.004**
**Multifocal tumor**	Yes vs. No	2.25	1.38–3.66	**0.001**
**Surgery type**	Biopsy	1		
	Partial	0.88	0.53–1.46	0.623
	Gross	0.53	0.33–0.86	**0.010**

Significance threshold: *p* < 0.05 ^1^ HR = Hazard Ratio, CI = Confidence Interval.

## Data Availability

The collected/analyzed data are not publicly available to protect patients’ privacy and comply with ethical requirements. Aggregated data supporting the study findings are available from the corresponding author upon a reasonable request.
